# *Plasmodium falciparum* Secretome in Erythrocyte and Beyond

**DOI:** 10.3389/fmicb.2016.00194

**Published:** 2016-02-19

**Authors:** Rani Soni, Drista Sharma, Tarun K. Bhatt

**Affiliations:** Department of Biotechnology, School of Life Sciences, Central University of RajasthanRajasthan, India

**Keywords:** plasmodium, secretome, cytoadherence, immune modulation, anti-malarial therapy

## Abstract

*Plasmodium falciparum* is the causative agent of deadly malaria disease. It is an intracellular eukaryote and completes its multi-stage life cycle spanning the two hosts viz, mosquito and human. In order to habituate within host environment, parasite conform several strategies to evade host immune responses such as surface antigen polymorphism or modulation of host immune system and it is mediated by secretion of proteins from parasite to the host erythrocyte and beyond, collectively known as, malaria secretome. In this review, we will discuss about the deployment of parasitic secretory protein in mechanism implicated for immune evasion, protein trafficking, providing virulence, changing permeability and cyto-adherence of infected erythrocyte. We will be covering the possibilities of developing malaria secretome as a drug/vaccine target. This gathered information will be worthwhile in depicting a well-organized picture for host-pathogen interplay during the malaria infection and may also provide some clues for the development of novel anti-malarial therapies.

## Introduction

World Health Organization report summarized that about 198 million cases and 0.58 million deaths occurred in year 2013 (World Health Organization [WHO], 2014). Amongst different species of *Plasmodium, P. falciparum* is the most dangerous and responsible for severe complexities during infection like multi-organ failure, cerebral malaria, coma, and death (Miller et al., 1994; Mendis and Carter, 1995). *P. falciparum* completes its life cycle spanning two alternate host, human, and mosquito. Within the human host, parasite undergoes series of developmental stages in the liver and erythrocytes (RBCs). The intra-erythrocytic cycle is found to be important as it is responsible for patho-physiology of the disease (Miller et al., 2002). Within the erythrocyte, the parasite proceeds through the different morphological stages such as ring, trophozoite, and schizont (Bannister et al., 2000; Florens et al., 2002). After completion of infection cycle, erythrocyte gets ruptured, and merozoites are released into the host bloodstream. The released merozoites initiate next round of erythrocytic cycle by infecting fresh erythrocytes (Gilson and Crabb, 2009). The survival of parasite inside the host cell is difficult, ascribed to which *Plasmodium* adapts various strategies to avoid the host immune response (Miller et al., 1994; Hisaeda et al., 2005). The strategies encompass the secretion of hitherto of proteins against infected RBC (iRBC) surface and beyond it into the host plasma (Singh et al., 2009). Infected erythrocyte surface remodeling is an example of such phenomena, involving the insertion of secreted proteins into the iRBC membrane (Deitsch and Wellems, 1996; Parker et al., 2004). Remodeling assists in cyto-adherence of iRBCs to the endothelial lining of blood vessels and thus averting them from splenic clearance (Newbold et al., 1999; Cooke et al., 2001; Rowe et al., 2009). Host immune modulation is another phenomenon involving the release of secretory proteins before or along with rupture of iRBCs. The released proteins interact with the components of the host immune system to provide conducive environment for merozoites before they invade new erythrocytes (Singh et al., 2009). The entire set of secreted proteins is known as ‘Secretome.’ These proteins are implicated in the processes essential for parasite survival such as host–parasite interactions and immune modulation (Ranganathan and Garg, 2009). Hence, in this review we will be focusing on parasite secretome and its significance in the malaria biology.

## Identification of Malaria Parasite’s Secretome

The export of secretory proteins from various species including *P. falciparum* has been thoroughly studied (van Ooij et al., 2008). Transport of secretory proteins follows a complicated route due to the presence of three membranes of parasite and vacuole inside the host cell (Martin et al., 2009). There must be a defined mechanism or recognizable sequence motifs behind such complex transport. Advent of bioinformatics tools facilitates the prediction of such conserved signal sequences responsible for the export and localization of the secretory proteins (Hiller et al., 2004; Marti et al., 2004, 2005).

The identification of sequence motifs necessary for export of parasite proteins is required for unearthing the complete secretome of the parasite. The first report proposed the presence of host targeting signal (HT motif) (Hiller et al., 2004) or *Plasmodium* export element (PEXEL motif) (Marti et al., 2004) in the sequence that is a requisite for the export of secretory proteins from parasitophorous vacuole (PV). The HT/PEXEL motif, present in more than 400 parasitic proteins, comprises short amino-terminal sequence, ‘R/KxLxE/Q’. The role of motif in the export of both soluble and surface-associated protein is determined by green fluorescent protein (GFP) and yellow fluorescent protein (YFP) assays involving the fusion of secretory proteins such as Knob associated histidine rich protein (KAHRP), *Pf*EMP-1 (with PEXEL like motif), Glycophorin binding protein (GBP130), and members of repetitive interspersed family (rifin). Mutation or truncation of the PEXEL motif interrupted the transport of proteins and rendered their accumulation in PV itself (Marti et al., 2004; van Ooij et al., 2008). Prior to the secretion of PEXEL containing proteins to destination, N-terminus is processed in endoplasmic reticulum (ER) by protease enzyme plasmepsin V (PMV), followed by N-acetylation of the cleaved product (Chang et al., 2008; Boddey et al., 2010; Russo et al., 2010). The decisive role of PMV in export of proteins is also demonstrated by identification of transition state (TS) inhibitor, WEHI-916 (Walter and Eliza Hall Institute of Medical Research). The WEHI-916 inhibitor is found to compete with the PEXEL containing substrate resulting in blocking of activity of PMV and ultimately lead to the cessation of parasite growth at the trophozoite stage. Knockdown studies of PMV further supported the inhibitory role on PMV. Indirect hindrance of export of PfEMP-1 and the loss of virulence and cytoadherence of iRBC has been observed due to inhibitory activity of WEHI-916 (Sleebs et al., 2014a,b). Recently, another inhibitor of PMV, WEHI-842 has been identified. The inhibitory role of WEHI-842 is assessed through the immunoblotting of GFP tagged PEXEL containing PfEMP-3. It is found to be more effective in comparison to WEHI-916 (Hodder et al., 2015). The presence of PEXEL motif leads to identification of first set of parasite secretome. However, HT/PEXEL is found missing in various secretory proteins (Lingelbach and Przyborski, 2006). Analysis of such sequences showed the presence of a hydrophobic stretch in the internal region of trans-membrane proteins which helps in crossing the Parasitophorous Vacuolar Membrane (PVM). Immune localization experiments and GFP-tagged approach investigated that first 20 amino acids of N-terminus along with hydrophobic residues in trans-membrane domain are common features of all PEXEL negative export proteins (PNEPs). Thus PNEPs have further expanded the secretome repertoire of the parasite (Spielmann et al., 2006; Spielmann and Gilberger, 2010; Heiber et al., 2013).

Apart from sequence-motifs based approach, other approaches are used to predict secretory proteins of *Plasmodium*. A Position-Specific Scoring Matrix (PSSM) profile based method is adapted that employs phylogenetic relationship derived through PSI–Blast against the non-redundant database. Based on these data, web server called ‘*Plasmodium* Secretory and Infected erythrocyte Associated Protein prediction’ (PSEApred) is developed to predict the secretory nature of *plasmodium* proteins (Verma et al., 2008). Similarly, the presence of N-myristoylation site, a cysteine S-palmitoylation site and some basic residues at N-terminus of parasite proteins are found to be responsible for the targeting to PVM and beyond (Gunaratne et al., 2000; Ma et al., 2012; Thavayogarajah et al., 2015; Wetzel et al., 2015). Together, both classical and non-classical path of secretion of proteins from parasite to the host cell have enhanced the secretome of the parasite. However, there is a possibility of appending more proteins to the growing secretome of parasite by identifying new signatures and patterns of secretion.

## Trafficking Pathway of Secretome

The protein containing PEXEL motif moves from ER after cleavage by PMV to the PV either in the form of vesicular cargo (Barnwell, 1989) or through the secretory apparatus (Hinterberg et al., 1994; Taraschi et al., 2003). Proteins that are not cleaved by PMV have been shown to bind with phosphatidylinositol 3- phosphate (PI3-P) in the ER and proceed for further trafficking pathway (Bhattacharjee et al., 2012). The PNEP proteins require transmembrane domain for their transport (Heiber et al., 2013). All the proteins in the PV are found to be exported through *Plasmodium* Translocon of Exported protein (PTEX) complex (de Koning-Ward et al., 2009; Beck et al., 2014; Elsworth et al., 2014). These proteins undergo unfolding for their translocation across PV (Charpian and Przyborski, 2008; Gehde et al., 2009; Gruring et al., 2012). *P. falciparum* has developed a membranous structure in the cytoplasm of the host cell called ‘Maurer’s cleft’. It is a secondary organelle and required for the export of the proteins involved in virulence, modification of host cell environment (Trager et al., 1966; Rudzinska and Trager, 1968) and for trafficking of membrane localized proteins (Przyborski et al., 2003; Lanzer et al., 2006; Mundwiler-Pachlatko and Beck, 2013). The exported proteins from PV are found to reside in the Maurer’s cleft (Haldar et al., 2002). Knock-down studies of Maurer’s cleft residing proteins like Membrane-Associated Histidine-Rich Protein (MAHRP1) and Skeletal binding protein-1 (SBP-1) proved its vitality in protein sorting (Epp and Deitsch, 2006; Maier et al., 2007; Spycher et al., 2008). In addition, most of the known secretory proteins including three antigenic families of parasite proteins (Stevor, Rifin, and Var) are localized in the Maurer’s cleft (Cheng et al., 1998) via PTEX export system (de Koning-Ward et al., 2009). PTEX export system, found exclusively in the genus *Plasmodium*, is responsible for the translocation of proteins targeted beyond the vacuolar membrane of the parasite (de Koning-Ward et al., 2009; Desai and Miller, 2014). It is a complex of five proteins including PTEX150, Heat shock protein 101 (HSP101), exported protein 2 (EXP2), PTEX88, and thioredoxin 2 (TRX2). The passage for directing proteins toward the cytosol of host erythrocyte is formed by EXP2 (de Koning-Ward et al., 2009). TRX 2 is found to be involved in unfolding of proteins destined to pass through the PTEX. Inhibition of HSP101 leads to the obstruction in protein export and eventually the accumulation of proteins such as Ring Infected Erythrocyte Surface Antigen (RESA), Ring Exported Protein 3 (REX3), Histidine Rich Protein-1 (HRP1), and KAHRP in parasitic compartment (Beck et al., 2014). It is observed that deletion of PTEX components prevent proteins from crossing PVM, resulting in interference of parasitic growth at the ring and trophozoite stage (Elsworth et al., 2014). However, mode of recognition between proteins to be exported and those to be retained by the PTEX complex still remains unclear. The mechanism of unfolding during protein export is also not defined and therefore it opens a new window of opportunity for scientists to explore and explain the facts related to PTEX system. In addition, the presence of this export system exclusively in *Plasmodium* genus makes it a captivating drug target (de Koning-Ward et al., 2009).

## Host Cell Remodeling

In order to make opportune environment within host, parasite makes substantial modifications in the host erythrocytes (Haldar and Mohandas, 2007). The modifications are predominantly mediated by secretion of parasite proteins across the PVM (Charpian and Przyborski, 2008; Maier et al., 2009; Goldberg and Cowman, 2010; Marti and Spielmann, 2013; Elsworth et al., 2014). The process of erythrocyte remodeling includes.

### Cytoadherence

To circumvent immune clearance in spleen, infected erythrocytes get adhered to endothelial wall, which is mediated through various cell adhesion molecules like ICAMs, CD36 on blood vessels (Gardner et al., 1996; Ho and White, 1999; Bhalla et al., 2015). Some events during adhesion process such as rosette formation with fresh erythrocyte (Udomsangpetch et al., 1989), auto-agglutination due to clumping of iRBCs and platelets (Pain et al., 2001) leads to severe disease pathologies (Rowe et al., 1995, 2002; Newbold et al., 1999). *P. falciparum* erythrocytic membrane protein-1 (*Pf*EMP-1) is major virulent factor present on surface of erythrocyte (Magowan et al., 1988; Chen et al., 1998). A study regarding transgenic lines of *P. falciparum* with altered *Pf*EMP-1 expression shows strong immune response targeted against *Pf*EMP-1 (Chan et al., 2012). Alteration in functioning of B-cells during parasite infection comprehends the interaction between cysteine-rich inter-domain region 1α (CIDRα) of *Pf*EMP-1 and B-cells. The complex formed causes the activation of NF-kB pathway resulting in functional impairment of B-cells (Simone et al., 2011). Multiple *Pf*EMP-1 proteins of *P. falciparum* bind to Fc portion of IgM (Jeppesen et al., 2015; Stevenson et al., 2015a) and found to be involved in rosette formation (Stevenson et al., 2015a,b). In addition to *Pf*EMP-1, sub-telomeric variant open reading frame (STEVOR) and RIFIN members also play decisive role in rosette formation (Cheng et al., 1998; Kyes et al., 1999; Niang et al., 2014). The antigenic variation of proteins allows the parasite to escape host immune response (Bull et al., 1998). A protruding structure on the surface of erythrocyte namely ‘knob’ is found to be essential in adhesion process of iRBCs (Crabb et al., 1997). Some proteins localized to knob interact with surface proteins of erythrocytes. Interactions include binding of KAHRP with ankyrin R and pro-coagulant glucosaminoglycans (Waller et al., 1999; Wickham et al., 2001; Rug et al., 2006; Weng et al., 2014) and binding of *Plasmodium* helical interspersed sub-telomeric domain (PHIST) to *Pf*EMP-1 (Oberli et al., 2014). In case of cerebral malaria, *Pf*14_075, member of PHIST family is found to be highly up-regulated and binds to human brain endothelial cell line (HBEC-5i). The study indicates its mantle in cyto-adherence (Claessens et al., 2012). Proteins such as erythrocyte membrane protein 3 (*Pf*EMP3), Mature parasite-infected Erythrocyte Surface Antigen (MESA; Lustigman et al., 1990), RESA, *Pf*EMP-1 (Sharma, 1997; Horrocks et al., 2005), KAHRP (Rug et al., 2006) and *Pf*EMP3 (Knuepfer et al., 2005) are involved in knob formation. Merozoites Surface Protein-1 (MSP-1), another knob protein, shows interaction with RBC surface proteins like Band 3 and Glycophorin A (GPA). A study of mouse model deficient in GPA-Band3 complex described the role of knob formation in cyto-adherence. (Goel et al., 2003; Baldwin et al., 2015).

### Membrane Permeability

Secretory proteins make astonishing alterations in the permeability of iRBCs membrane for ions and nutrient exchange. (Homewood and Neame, 1974; Ginsburg et al., 1983; Kutner et al., 1983). The presence of ion channels such as *Plasmodium* Surface Anion Channel (PSAC) is responsible for induction of drug resistance. The identification of structural composition of this complex would contribute to better understanding of pathogenic interaction and drug resistance mechanism and therefore suggested for therapeutic intervention (Lisk et al., 2008; Desai, 2012). Cytoadherence-linked antigen3 (Clag3) protein, found on the host membrane is appraised to be associated with PSAC in ion and nutrient transport through channels (Nguitragool et al., 2011; Pillai et al., 2012; Sharma et al., 2015). Secretory proteins involved in regulation of net flux of Na^+^, K^+^, and other ions are on the focus (Kirk, 2015). For instance, P-type ATP4 (*Pf*ATP4), regulating the transport of Na ^+^ ions is contemplated as a potential drug target (Spillman et al., 2013). Membrane permeabilization is found to be a necessary event for egress of parasites from iRBCs. Cysteine proteases have been shown to play cardinal role in rupture of erythrocyte membrane for the release of parasite (Hadley et al., 1983; Dluzewski et al., 1986; McKerrow et al., 1993; Raphael et al., 2000; Lee and Fidock, 2008). One of the members of this class, falcipian 2 is responsible for the cleavage of ankyrin and protein 4.1 of erythrocytic cytoskeleton (Dua et al., 2001). It has been evident through the gene disruption studies that expression of *Plasmodium* perforin like protein 2 (PPLP2) is paramount for membrane permeabilization during the gametocyte release from infected erythrocytes. It had been illustrated that gametocytes are unable to release from PPLP2 (-) lines of parasite, thereby reducing the transfer of gametocyte to vector (Wirth et al., 2014). MSP-1 has also been demonstrated to interact with host cytoskeleton spectrin causing the membrane destabilization and thereby enabling the release of merozoites from iRBC (Das et al., 2015).

### Membrane Rigidity

Apart from cytoadherence, membrane rigidness or loss of deformability is also responsible for the sequestration of iRBCs (Bull et al., 1998). It has been clarified that knobs are liable for causing stiffness and hardening of the iRBCs (Zhang et al., 2015). Deformability of parasitized RBC is reduced due to association of RESA with spectrin (Mills et al., 2007). *Pf*332 exported on the membrane is directly involved in membrane rigidity and adhesion (Glenister et al., 2009). The KAHRP along with the membrane skeleton imparts rigidity to infected cell and will eventually obstruct blood flow (Waller et al., 1999; Pei et al., 2005). PHIST protein increases membrane rigidity by binding to membrane skeleton (Parish et al., 2013). Thus, it can be surmised that proteins responsible for rigidity are directly linked to virulence, providing an evidence for secretome in establishment of infection.

## Secretory Protein Exported Beyond the Erythrocyte

Most of the data reported with respect to secretome is related to proteins secreted into the erythrocytes cytosol or membrane. Interestingly, some proteins, which are not restricted to iRBCs membrane rather squeeze out from iRBCs membrane and get secreted out. First experimental evidence (Singh et al., 2009) identified secretion of 27 novel proteins beyond the erythrocyte membrane before it gets ruptured. Immune localization and immune electron microscopic studies confirmed the secretion of proteins beyond iRBC (Singh et al., 2009). Some of them are functionally characterized. The protein containing Sel-1 functional domain is found to be involved in regulating ‘Notch signaling pathway’ which in turn has been hypothesized to influence the T cell differentiation (Grant and Greenwald, 1996; Singh et al., 2009). In most protozoan parasites, to evade host immune response, common mechanism includes altered T-helper cell differentiation (Zambrano-Villa et al., 2002; Rodrigues et al., 2014). Some proteins, closely associated with highly polymorphic genes, contribute to antigenic determinants of parasite (Singh et al., 2009). Secretory protein with LCCL (Limulus clotting factor C) domain, conserved across apicomplexan parasite, assumed to have role in immune evasion mechanism, (Claudianos et al., 2002; Dessens et al., 2004), defense mechanism and shows binding with lipid A of lipopolysaccharides. CRISPLD2 (Cysteine-Rich Secretory Protein LCCL Domain containing 2), an example of LCCL domain containing protein, has an anti-inflammatory function and is related to disease pathology (Vásárhelyi et al., 2014).

Sequence similarity studies suggested that some proteins viz, virulent immuno-reactive protein (specific to bacteria and virus), PFB0765w (uncharacterized protein), rhoptry neck protein (RON4), moving junction protein and MAL13P1.39 (uncharacterized protein) are involved in modulation of host immune response (Singh et al., 2009). Domain analysis demonstrated the presence of extracellular domain responsible for the interaction with other proteins, speculative of being involved in host–parasite interactions. [**Table 1**: Domains identified by CDD (Conserved Domain Database), NCBI]. It is depicted through flowcytometery and confocal microscopy that translationally controlled tumor protein (TCTP) analog released by *Plasmodium* in host serum is responsible for release of histamine and IL-8 from basophils and eosinophils, respectively, (MacDonald et al., 2001) and reduction in B-cell immune response. In another study, a canonical tyrosyl-tRNA synthetase (*Pf*TyrRS) from *Plasmodium* is evidenced to be secreted out from the iRBCs and involved in non-canonical function of immune cell binding and modulation (Bhatt et al., 2011). Likewise in other intracellular pathogens such as *Mycobacterium tuberculosis* immune modulation ability is found in secretory proteins (Giacomini et al., 2001). During infection, secretome is also charged for causing alteration in functioning of antigen-presenting cells and dendritic cells (Sacks and Sher, 2002; Langhorne et al., 2004; Millington et al., 2006; Sponaas et al., 2006; Teirlinck et al., 2015). The presence of proteins on the surface or in secretion implicate their role in host–parasite interactions and probably in immune modulation for better survival of parasite and it would be fascinating to have information related to the ‘Interactome’ of the secretory proteins. *In-silico* knock-out studies and graphical analysis of protein–protein interaction network (PPIN) explored newer approach in order to identify the interacting partners vital to parasite during host–parasite interaction (Bhattacharyya and Chakrabarti, 2015). Nevertheless, some more studies are required to understand the role of secretory proteins in regulating host pathways. The role of secretory proteins of the parasite could have larger impact on malaria biology. Besides available knowledge, there is a need to identify signature motif or pattern responsible for secretion of proteins outside iRBCs. The identification of marker responsible for the localization of proteins to infected erythrocyte membrane and their export will be highly beneficial in interaction studies. There is a requirement of classifying secretome in terms of cellular localization and expression during developmental stages of parasite in order to understand its role in better way. It would be interesting to explore trafficking pathway of protein exported beyond the iRBCs membrane.

**Table 1:** Some secretory protein exported out from the iRBC.

Sr. No	Gene name	Domain description/protein name	Reference
1	MAL7P1.138	_	Singh et al., 2009
2	MAL8P1.126	Serine protease DegP	Singh et al., 2009
3	MAL13P1.24	–	Singh et al., 2009
4	MAL13P1.39	_	Singh et al., 2009
5	*PF*07_0074	_	Singh et al., 2009
6	*PF*07_0086	Uncharacterized protein with domain 1. TATA element modulatory factor 1 2. DNA repair protein RAD18	Singh et al., 2009
7	*PF*07_0113	_	Singh et al., 2009
8	GBP-*PF*10_0159	1. Glycophorin-binding protein	Singh et al., 2009
9	*PF*10_0318	1. Uncharacterized / ACR, YagE family domain	Singh et al., 2009
10	*PF*10_0380	Trophozoite antigen R45, putative	Singh et al., 2009
11	*PF* EP *PF*11_0139	Protein tyrosine phosphates	Singh et al., 2009
12	RON4 *PF*11_0168	Moving junction protein	Singh et al., 2009
13	TKL-2 *PF*11_0220	Protein Kinase	Singh et al., 2009
14	*PF*11_0324	Uncharacterized protein	Singh et al., 2009
15	*PF*11_0369	Uncharacterized protein	Singh et al., 2009
16	*PF*11_0381	Subtilisin-like protease 2	Singh et al., 2009
18	*PF*13_0198	Reticulocyte-binding protein 2 homolog a	Singh et al., 2009
19	*PF*14_0462	SEL-1 protein, putative	Singh et al., 2009
20	CCP1 *PF*14_0723	LCCL domain containing protein CCP1	Singh et al., 2009
21	*PF*A018w	1. L-seryl-tRNA(Sec) kinase, 2. Predicted nucleotide kinase	Singh et al., 2009
22	*PF*B0190c	Conserved *Plasmodium* protein with domain 1. Sel1-like repeats 2. TPR repeat, SEL1 subfamily	Singh et al., 2009
23	*PF*B0315w	Uncharacterized protein *PF*B0315w	Singh et al., 2009
24	*PF*b0465c	Monocarboxylate transporter, putative with domain 1. The Major Facilitator Super family (MFS) 2. Oxalate/formate antiporter family transporter. 3. Monocarboxylate transporter	Singh et al., 2009
25	*PF*B0655c	Conserved *Plasmodium* protein	Singh et al., 2009
26	*PF*B0750w	Vacuolar protein-sorting protein VPS45, putative	Singh et al., 2009
27	*PF*B0765w	Uncharacterized protein *PF*B0765w with domain 1. Chromosome segregation ATPases 2. Myosin class II heavy chain [Cytoskeleton]	Singh et al., 2009
28	*PF*E0245c	Uncharacterized protein with domain 1. Dos2-interacting transcription regulator of RNA-Pol-II 2. DNA repair/transcription protein Mms19 3. Ultrahigh sulfur keratin-associated protein	Singh et al., 2009
29	*PF*E0440w	Uncharacterized	Singh et al., 2009
30	*PF*L0030c	Erythrocyte membrane protein 1 (*PF*EMP1)	Singh et al., 2009
31	*PF*I1150w	HRP II/I II domain	Singh et al., 2009
32	*PF*L2405c	Chromosome segregation protein SMC (structural maintenance of chromosomes) *PF*G377 protein	Singh et al., 2009
33	*PF*TyrRS	Tyrosyl-tRNA synthetase	Bhatt et al., 2011
34	*PF*TCTP	*Plasmodium falciparum* translationally controlled tumor protein	MacDonald et al., 2001; Calderon-Perez et al., 2014

## Secretome as Potential Drug/Vaccine Target

The intracellular parasite adapts different strategies for protein export in order to survive in host environment. As the secretome is intimately associated with disease pathology and parasite survival is reliant on them, any interference in the secretory pathway or inhibition of secretory proteins itself would jeopardize the parasite. In addition, utilization of information of secretome available shall provide clues to certain strategies involved in host–parasite interaction at molecular level (Ranganathan and Garg, 2009). The function of these proteins can be annotated by comparing with homologous protein of known function in other organisms. Homology modeling of secretory proteins could also provide a starting point for the lead identification in the process of drug development. Vaccine and drug development against the secretory protein is in progress in various other pathogens like *H. pylori* (Lower et al., 2008), Helminths parasite (Hewitson et al., 2009) etc. Till date, various parasite proteins involved in secretory pathway have been characterized and may be critical in anti-malarial drug targeting such as inhibition of PTEX complex. Another important drug target capturing the interest in context of drug development area is PMV (plasmepsin V). Indispensability of PMV in virulence, cytoadherence, and parasitic growth makes PMV an attractive anti-malarial drug target (Sleebs et al., 2014a,b). Structural determination of PMV–WEHI 842 inhibitor complex provides an insight for interaction between active site residue and inhibitor. This study paves the way for developing potent anti-malarial by blocking export machinery of parasite (Hodder et al., 2015).

Taken together, the functional characterization of secretory proteins and proteins involved in their export, implicated in knob formation, involved in trafficking pathway, or those involved in host pathogenic interaction and invasion of host immune system, are all indispensible for parasite survival or pathogenicity. Therefore, complete investigation and characterization of secretome may provide us better understanding to get effective therapies for malaria disease. Allelic replacement and GFP tagging revealed the importance of PMV in protein export and parasite survival thus making it an attractive target for anti malarial drugs.

## Conclusion

Export of secretory parasite proteins into host cytoplasm will lead to apprehension of host cell functions required for parasite growth and survival by modulating crucial phenomena of malaria biology such as immune evasion and virulence. Deep understanding and investigation of role played by malaria secretome will be not only beneficial in deciphering host–pathogen interactions but it may also lead to better therapeutic intervention for malaria disease.

## Author Contributions

RS and DS provided data and TB wrote the manuscript.

## Conflict of Interest Statement

The authors declare that the research was conducted in the absence of any commercial or financial relationships that could be construed as a potential conflict of interest.
